# APSD up to 100 kHz dataset measured in the IPEN/MB-01 research reactor facility

**DOI:** 10.1016/j.dib.2020.106609

**Published:** 2020-11-29

**Authors:** Diogo Feliciano dos Santos, Adimir dos Santos, Ricardo Diniz

**Affiliations:** Nuclear Engineering Center, Nuclear and Energy Research Institute (IPEN), 2242 Prof. Lineu Prestes Avenue, 05508-000, Cidade Universitária, São Paulo, SP, Brazil

**Keywords:** Zero power reactor noise, APSD measurements, IPEN/MB-01 reactor, IPEN/MB-01 Correlator, Subcritical experiments

## Abstract

The data presented in this work are from the very accurate reactor noise measurements performed in the IPEN/MB-01 research reactor facility. The quantity inferred from the measured data was the Auto Power Spectral Density (APSD) with the frequency range extended up to 100 kHz. The core configuration considered a short version of the IPEN/MB-01 core, consisted of a 26 × 24 rectangular array of fuel rods with control banks totally withdrawn. The measured reactivity excess for this configuration was equal to (10 ± 3) pcm. The subcriticality was reached by poisoning the reactor water with boric acid (H_3_BO_3_) in the concentrations of 286.8 and 578.6 ppm of natural boron. The main goal of these experiments was to test subcritical configurations with uniform poisoning. The average temperature for the experiment with 286.8 ppm of natural boron was (19.82 ± 0.37) °C and that for the 578.6 ppm was (19.89 ± 0.09) °C. The core was driven by the ^241^Am-Be start-up source (∼1.0 Ci) of the facility placed in the reflector. The APSD (in units of count^2^/Hz) was inferred employing the IPEN/MB-01 Correlator. The basic measured data arise from the pulses of two ^3^He Centronic detectors placed in the reflector region.

## Specifications Table

**Table:** 

Subject	Nuclear energy and engineering
Specific subject area	Auto Power Spectral Density (APSD) measurements
Type of data	CSV files
How data were acquired	Two ^3^He Centronic detectors count the neutrons born in the IPEN/MB-01 nuclear reactor core. The detector pulses were sent through the instrumentation, consisting of preamplifiers, amplifiers, Single-Channel Analyzer (SCA), Input Logic Unit, a computer with a Multichannel Scaler (MCS), and a program written in LabView language to generate the APSD spectral densities.
Data format	Raw Analyzed
Parameters for data collection	The core configuration considered a short version of the IPEN/MB-01 nuclear reactor core consisted of a 26 × 24 rectangular array of fuel rods with the control banks totally withdrawn. Subcriticality was reached by poisoning the reactor water with boric acid (H_3_BO_3_) in the concentration of 286.8 and 578.6 ppm of natural boron. The core/moderator temperature for the subcritical experiments was required to be (20.00 ± 0.50) °C for all measurements. The core was driven by an external source placed in the reflector.
Description of data collection	Two ^3^He Centronic detectors were placed in the reflector region of the nuclear reactor core for the data acquisition. The detector neutron pulses were formatted and amplified by preamplifiers and amplifiers. Subsequently, they were discriminated from the γ-radiation through the Lower Level Discriminator of the Single-Channel Analyzer (SCA). Negative logical pulses were generated in the output of the single-channel. The negative logic pulses were summed in a logic OR employing an Input Logic Unit. A Multichannel Scaler (MCS) board registered the time intervals between a trigger signal and the subsequent logical pulses. A program written in LabView 5.1 and supported by the C/C++ language makes the data processing. The MCS transforms the pulses from the detectors into a time spectrum, which represents the count rates in the detector.
Data source location	Institution: Nuclear and Energy Research Institute (IPEN) City/Town/Region: São Paulo Country: Brazil Primary data sources: The primary data are the ^3^He detector APSD in the frequency domain. They are in the files named spectra with the number of the acquisition. For the 286.8 ppm natural boron case there are 12 spectra files, while for the 578.6 ppm case there are 163.
Data accessibility	Data are in public repository. Repository name: GitHub Direct URL to data: https://github.com/diogofs6086/APSD_Data
Related research article	D. F. dos Santos and A. dos Santos, Zero-power noise up to 100 kHz in the IPEN/MB-01 research reactor facility, approved for publication at Annals of Nuclear Energy, 2020. https://doi.org/10.1016/j.anucene.2020.107974

## Value of the Data

• The reported data provide insights to infer several significant aspects of the kinetic behavior of thermal compact reflected-core systems.
• Currently, the experimental reactor data are scarce because the number of active nuclear research reactors has decreased during the years. Therefore, the experimental data of this approach can benefit the reactor physics community.
• The experimental data can be useful for the development of new kinetic models for the reflected core reactors, and from that to infer relevant quantities of the nuclear reactor physics field, such as subcritical reactivity, neutron lifetimes, generation times, and prompt neutron decay constants in the core and reflector regions.

## 1 Data Description

The measurement data are saved in text files, named “spectra” with the number of the acquisition. There was an experiment with 286.8 ppm of natural boron and another with 578.6 ppm. The experimental data are in a GitHub repository, named “APSD_Data”, indicated in the field “Data accessibility”. The repository has a folder for each experiment, which contains scripts in Python and R languages for anyone who wants to run and creates the average APSD files in comma-separated-values (CSV) format and the graphics. Each folder has another folder, named “Raw_data”, containing the “spectra” files. These files have four columns and 31250 rows. The length of the row is given by the number of the time channels for the one-side spectrum chosen for the experiments. The first file column corresponds to the number of the time channels for the one-side spectrum, that must be transformed to the frequency f in Hertz (Hz) as

(1) $$f=\frac{C_{1}}{DC_{2}},$$

where $C_{1}$ is the first column of the file, $C_{2}$ is the number of the time channels for the two-side spectrum, which is the double of the number of the one-side spectrum, and D is the dwell time (the time bin). The second column has the values of the APDS in the units of count^2^/Hz. The third and fourth columns have only zeros, meaning that they were not utilized and can be dismissed. In this kind of measurement by the IPEN/MB-01 Correlator, the first two rows must be deleted because the acquired values are very discrepants.

The 286.8 ppm case has 12 measurements (files) and the 578.6 ppm 163. From that files were calculated the mean of the second columns to obtain an average APSD for each case. The uncertainties of each measurement are given by $1/\sqrt{N}$, where N is the number of count rates performed. It has the name of “averages”, which can provoke a little confusion, but when the words “averages”, in the plural, appears in the text, it means the N value. The uncertainty of the average APSD is given by

(2) $$\sigma =\frac{1}{n}\sum_{i=1}^{n}\sqrt{\frac{A_{i}^{2}}{N}},$$

where n is the number of measurements and A is the second column of the files (APSD columns).

After the transformation to obtain the average APSD data files for the 286.8 and 576.8 ppm of natural boron cases, the APSD values can be plotted as a function of the frequency. The plotted curves start in lower frequencies with a first plateau, decrease, show another plateau, and increase in value at the higher frequencies. The reactivities in units of dollar calculated from MCNP6 [1] in conjunction with the ENDF/B-VII.0 library were −8.95 $ and–17.43 $ for 286.8 and 578.6 ppm of natural boron, respectively. A MCNP sample input is on the reference [2], but it needs the modification of the moderator boron concentration. The effective delayed neutron fraction from IPEN/MB-01 reactor is β_eff_ = (750 ± 5) pcm [3].

## 2 Experimental Design, Materials and Methods

A series of subcritical experiments were conducted at the IPEN/MB-01 research nuclear reactor facility, located in the city of São Paulo, Brazil, in 2018. This set of subcritical experiments [4,5] consider the water tank (moderator) doped with soluble boric acid to get the subcritical configurations. The evaluated square core configuration consists of uniform lattices of stainless-steel-clad cylindrical fuel rods immersed in light water doped with soluble boric acid. The pitch of the rods is 15.0 mm, which is close to the optimal pitch (maximum k_∞_). The configuration core of the IPEN/MB-01 reactor employed throughout the experiments was a reduced version of its standard configuration, which was a 26 × 24 fuel rod array. The core was driven by the ^241^Am-Be start-up source (∼1.0 Ci) of the facility placed in the reflector. The subcritical state was reached by totally removing the control banks and by inserting soluble boric acid in the moderator. The intended core/moderator temperature for the subcritical experiments was (20.00 ± 0.50) °C for all configurations.

**Fig. 1:**
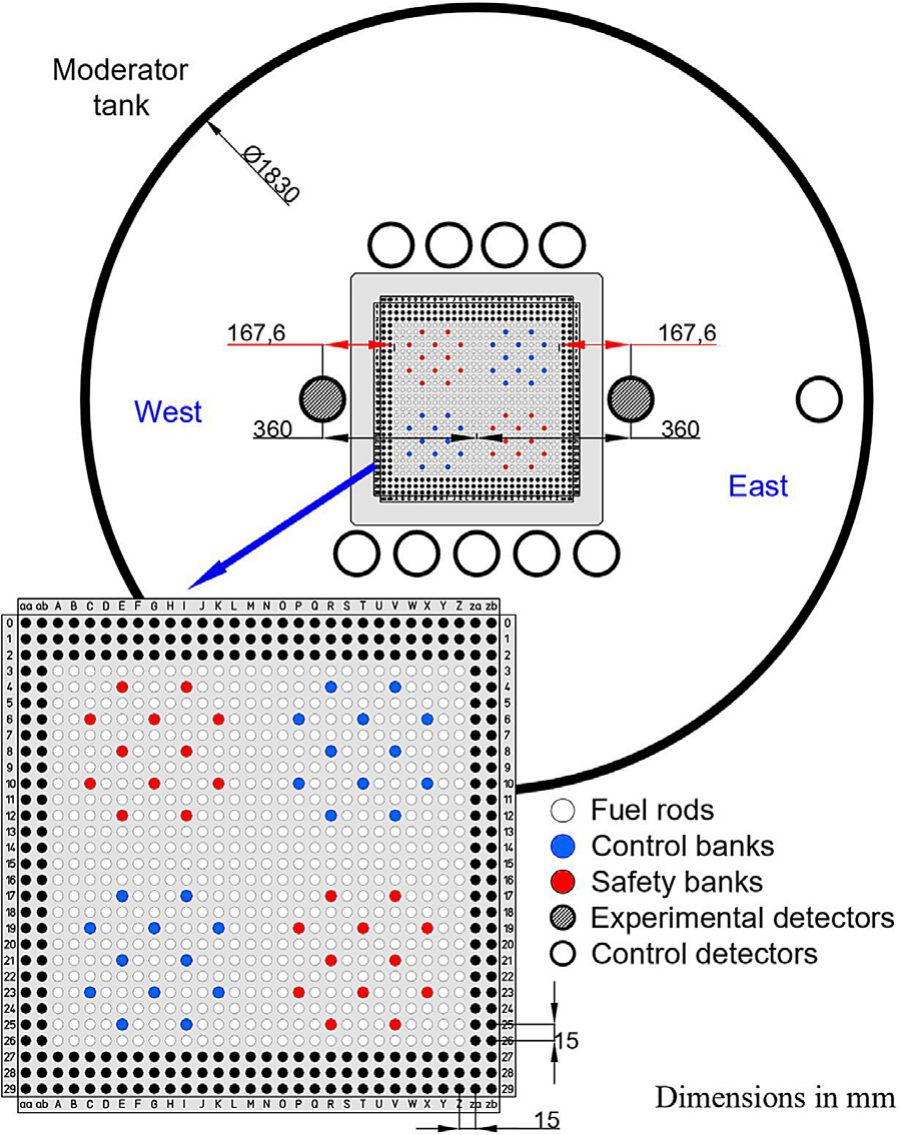
Radial schematic diagram of the moderator tank.

**Fig. 2:**
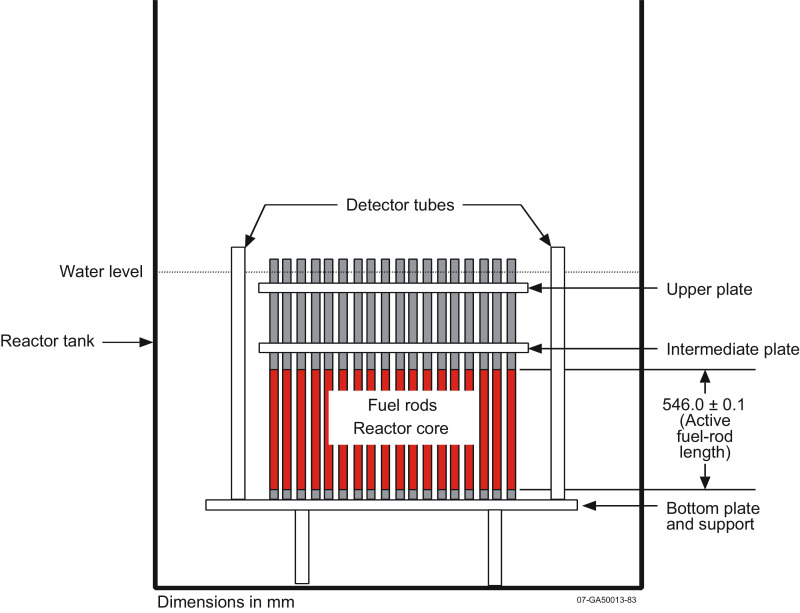
Axial schematic diagram of the moderator tank.

A complete description of the IPEN/MB-01 core is elsewhere [2]. Here, the description is mainly restricted to the details of the subcritical configuration employing soluble boric acid in the moderator water and its interface to the measurement systems. The IPEN/MB-01 research reactor is a nuclear facility that, excluding fuel burnup and heat transfer in the core, allows simulation of several characteristics of a power reactor on a reduced scale. Demineralized light water is used as a moderator. Fig. 1, Fig. 2 illustrate respectively the radial and axial views of the experimental configurations employed in this work. The IPEN/MB-01 reactor core is composed of an array of fuel rods. Each fuel rod consists of a cladding (SS-304), UO_2_ pellets (enriched to 4.3486%), alumina (Al_2_O_3_) pellets, a spring (Inconel-600), spacer tube (SS-304), and top and bottom plugs (SS-304). During normal operations, the water level stays at least 450 mm above the top of the UO_2_ column. Laterally, the water thickness is larger than 600 mm, and below the active, core the water is at least 530 mm deep.

The two control rod banks of the IPEN/MB-01 reactor are located diagonally opposite each other in the core, as well as the safety rod banks, as indicated in Fig. 1. Each control bank is composed of 12 rods of an alloy of Ag-In-Cd wrapped in a SS-304 cladding. The mass percentage of the alloy is composed of 80% of Ag, 15% of In, and 5% of Cd. If the control rods are fully withdrawn, they will be completely removed from the active core and their bottom part will be at the top of the active fuel length. Each safety bank is also composed of 12 rods, except that the neutron absorber is a compact powder of Boron Carbide (B_4_C). The safety banks by design criteria are kept at a removal position of 35 % above the active fuel length. Therefore, when the safety banks are in a totally withdrawn position, they have a negligible impact on the neutron multiplication of the system.

The IPEN/MB-01 reactor possesses a heating/cooling and water-circulating system that allows precise control of the temperature in the reactor core. The temperature of the reactor core can be set anywhere in the range from 7 °C to 90 °C. The core temperature was monitored through a set of 12 thermocouples strategically located in the reactor core. This 1.6 mm diameter thermocouples are made of an alloy of Cu-Ni (55 wt % Cu and 45 wt % Ni).

The experimental configuration comprises a rectangular 26 × 24 fuel rod array, as shown in Fig. 1. Relative to the standard 28 × 26 fuel rod array configuration, the small one considered the removal of the outer row and the outer column of fuel rods in each face, i.e., 104 fuel rods. Almost all the reactivity excess was removed from the core with no boron in the moderator by totally withdrawn the control and safety banks, which measured (10 ± 3) pcm. This was an ideal reference configuration since the control bank effect is practically eliminated and the reactivity induced by the addition of the soluble boron acid in the moderator water is related to this slightly supercritical configuration.

The boron solutions were produced in the following way. The facility has a boron dosing tank, which is a small tank specially designed to prepare the boron solution. This tank has electric heaters to increase the water temperature and a mix stirrer. Both tools were used to homogenize the boron acid in the water. Initially, the boron dosing tank was filled with water from the storage tank and this water was heated to 60 °C employing the electric heaters. After that, the desired amount of boric acid (H_3_BO_3_) powder was inserted into the boron dosing tank. Next, the mixture homogenization started by turning on the mix stirrer for 30 min, and then the boron solution was returned to the storage tank. To guarantee the mixture homogenization, the boron solution in the storage tank was pumped to the moderator tank up to nearly the ordinary level of operation, and the water circulation system was turned on for about 2 h. After that, the boron solution was discharged to the storage tank. The last three steps were repeated several times to guarantee the homogenization was achieved. Samples of the boron solution were collected and sent to the chemical laboratories of the University of Sao Paulo. This whole pattern was repeated for all boron solutions mixed in the IPEN/MB-01 reactor.

Seven boron solutions were mixed in the moderator tank, producing a natural boron content in the water. The natural boron in the moderator was measured in the Central Analytic of the Chemistry Institute of the University of São Paulo. The equipment employed was the ICP OES, Radial (Arcos model), manufactured by Spectro Company. The uncertainty in the measurement was 10 ppm, corresponding to the equipment accuracy and the process employed to obtain the boron content. These data are given in Table 1.

**Table 1:** Measured natural boron content in the moderator.

Experiment number	Boron content (ppm)
1	47.9 ± 10
2	88.9 ± 10
3	136.5 ± 10
4	185.1 ± 10
5	245.2 ± 10
6	286.8 ± 10
7	578.6 ± 10

The first six experiments were approved by the ICSBEP (International Criticality Safety Benchmark Evaluation Project) as international subcritical benchmarks [2]. For these experiments, the APSD and CPSD were acquired up to 4 kHz, and each 1 ppm of natural boron in the water was equivalent to about 23.6 pcm of negative reactivity [4]. This high value of specific reactivity is due to the neutronic characteristics of the IPEN/MB-01 core, which has its pitch very close to the optimum pitch (maximum $k_{\infty }$). The work presented here employed 6^th^ and 7^th^ experiments, but the frequency range of the inferred APSD was extended up to 100 kHz.

The experiments 6 and 7 employed the two ^3^He Centronic detectors described in Table 2. They were placed 167.6 mm away from the outermost fuel rods of the reactor, inside the detection channels and at the same level as the upper surface of the bottom grid plate. Due to the high subcritical level, the two most sensitive detectors available in the IPEN/MB-01 facility were selected. They had 100 cm height sensitive and covered the entire active height given by the fuel rods, exceeding it by 42.5 cm approximately. The experimental detectors were symmetrically located in the reflector region, as shown in Fig. 1, to get the neutron counts for the IPEN/MB-01 Correlator. The counts were then summed to get better statistics in the measurements.

**Table 2:** Detector specifications.

**Serial number**	8739	8740
**Model**	100He3/152/38HS	100He3/304/38HS
**Sensivite diameter (mm)**	36.32	36.32
**External diameter (mm)**	38.1	38
**Sensitive length (mm)**	1000	1000
**External diameter (mm)**	1034.8	1034.8
**Pressure (atm)**	2	4
**Operating voltage (kV)**	1.0	1.2
**Sensitivity (cps/nv)**	202	297
**Core face**	West	East

**Table 3:** Operation number, date, acquisition number, and average temperatures.

Operation number	Date	Natural boro concentration (ppm)	Acquisition Number	Average Temperature (°C)
3639	05/14/2018	286.8	1 through 4	19.99 ± 0.68
3641	05/16/2018	286.8	5 through 12	19.66 ± 0.27
3642	05/18/2018	578.6	1 through 5	19.92 ± 0.66
3644	05/21/2018	578.6	6 through 15	19.75 ± 0.28
3645	05/22/2018	578.6	16 through 22	19.75 ± 0.62
3646	05/23/2018	578.6	23 through 32	19.61 ± 0.51
3647	05/24/2018	578.6	33 through 43	19.65 ± 0.29
3648	05/25/2018	578.6	44 through 60	20.12 ± 0.26
3650	05/30/2018	578.6	61 through 75	19.62 ± 0.26
3651	06/04/2018	578.6	76 through 82	19.94 ± 0.50
3652	06/05/2018	578.6	83 through 100	19.74 ± 0.16
3653	06/06/2018	578.6	101 through 116	19.72 ± 0.15
3654	06/07/2018	5786	117 through 130	19.82 ± 0.13
3655	06/08/2018	578.6	131 through 148	20.38 ± 0.07
3656	06/11/2018	578.6	149 through 155	20.19 ± 0.14
3657	06/12/2018	578.6	156 through 163	20.51 ± 0.14

The data acquisition and processing system employ the following steps. Neutron pulses from the detectors were formatted and amplified by preamplifiers and amplifiers with the shaping time set to 2 μs. Subsequently, they were discriminated from the γ-radiation through the Lower Level Discriminator of the Single-Channel Analyzer (SCA). Negative logical pulses were generated in the output of the single-channel (standard NIM fast negative) with 25 ns width and -5 V of amplitude on 50 Ω impedance. Since the subcritical level was high for the two boron diluted cases considered here, the negative logic pulses were summed (merged) in a logic OR (*X* = A + B) employing an Input Logic Unit. A Multichannel Scaler (MCS) board registered the time intervals between a trigger signal and the subsequent logical pulses. The dwell time chosen for the MCS board provided the maximum frequency to be analyzed, and the number of channels gave the corresponding frequency resolution. The whole system of acquisition and signal processing is called IPEN/MB-01 Correlator, and its program is written in LabView 5.1 language (Laboratory Virtual Instrument Engineering Workbench).

The MCS transforms the pulses from the detectors into a time spectrum, which represents the detector count rates. Once the detector count rates are obtained, the level DC (average count rate) is removed from the count rates. This is like an electronic filter, however, performed by a software. Finally, the APSD is constructed consistently to that employed in current mode by the DSA (Agilent 35670A Dynamical Signal Analyzer) [6] as:

(3) $$APSD=\frac{\sum_{i=1}^{N}FFT{\left(CR\left(t\right)\right)}_{i}\cdot FFT{{\left(CR\left(t\right)\right)}_{i}}^{*}}{NB},$$

where FFT is the Fast Fourier Transform of CR(t) (counts), CR(t) is the count rate minus DC at time t (in units of cps), N is the number of averages, B is the bandwidth (Hz), and the symbol (*) represents the complex conjugate of FFT(CR(t)).

In this work, the dwell time was set at 5 µs, the bandwidth was 4.8 Hz, the number of channels, in the time domain, was set to be 62500 (double-sided spectrum), which resulted in a resolution of 3.2 Hz in the frequency domain, and a frequency domain from 9.6 Hz up to 100 kHz (single-sided spectrum).

The APSD unity arising from Eq. (3) is count^2^/Hz. Experimentally, the IPEN/MB-01 Correlator is very similar to the one developed by Kitamura [7] although there are some differences in the CPSD magnitude and its unity. The APSD derived from the IPEN/MB-01 Correlator preserves the correct units and magnitude.

The APSD measurements performed by the IPEN/MB-01 Correlator considered 3000 averages (the number of count rates) in each acquisition. Each acquisition took about 35 minutes to be completed. The average count rates (cps) were, respectively, (923 ± 11) cps and (198.2 ± 1.1) cps for the 286.8 and 578.6 ppm of natural boron cases. The number of acquisitions was, respectively, 12 and 163 for the 286.8 ppm and 578.6 ppm cases. The 578.6 ppm case has more acquisitions due to its high degree of subcriticality and lower detector counts.

The average core temperature monitored by the thermocouples for the 286.8 ppm natural boron case was (19.82 ± 0.37) °C and for the 578.6 ppm case was (19.89 ± 0.09) °C. Table 3 shows the temperature acquisition history of experiments 6 and 7.

## CRediT Author Statement

Diogo Feliciano dos Santos: Conceptualization, Methodology, Data curation, Formal analysis, Writing- Original draft preparation, Writing - Review & Editing. Adimir dos Santos: Conceptualization, Methodology, Data curation, Formal analysis, Resources, Writing- Original draft preparation, Writing - Review & Editing, Supervision. Ricardo Diniz: Formal analysis.

## Declaration of Competing Interest

The authors declare that they have no known competing financial interests or personal relationships which have, or could be perceived to have, influenced the work reported in this article.

## References

[1] Goorley J.T., James M.R., Booth T.E., Brown F.B., Bull J.S., Cox L.J., Durkee J.W., Elson J.S., Fensin M.L., Forster R.A., Hendricks J.S., Hughes H.G., Johns R.C., Kiedrowski B.C., Mashnik S.G., Mckinney G.W., Pelowitz D.B., Prael R.E., Sweezy J.E., Waters L.S., Wilcox T.A., Zukaitis A. (2013). MCNP6 User's Manual Version 1.0.

[2] dos Santos A., de Souza G.S., dos Santos D.F., Andrade D.A., Rocha M.O., Galia M.V.C., Diniz R., Jerez R. (2019). Loading configurations of the IPEN/MB-01 reactor with soluble boric acid in the moderator. Int. Handb. Eval. Crit. Saf. Benchmark Exp..

[3] dos Santos A., de A. Silva G.S., Fanaro L.C.C.B., Yamaguchi M., Jerez R., Diniz R., Carneiro Á.L.G., Kuramoto R.Y.R., Mendonça A.G., Fuga R., de R., Maeda M., Mura L.F.L. (2006). IPEN(MB01)-LWR-RESR-001 CRIT-BUCK-SPEC-REAC-COEF-KIN-RRATE-POWDIS REACTOR: reactor physics experiments in the ipen/mb-01 research reactor facility. Int. Handb. Eval. React. Phys. Benchmark Exp., NEA/NSC/DOC/(2006)1.

[4] dos Santos A., de Souza G.S., dos Santos D.F., de Andrade e Silva G.S. (2019). Subcritical boron experiments in the IPEN/MB-01 reactor. European Nuclear Society (Ed.), RRFM Eur. Res. React. Conf..

[5] Santos A., dos Santos D.F., De Souza G.S., Loureiro C.D. (2018). Reactor noise at high frequencies and subcritical reactivity inference. Fourth Int. Conf. Phys. Technol. React. Appl., Marrakech.

[6] T.I. Keysight, Keysight 35670A Dynamic Signal Analyzer, (2017). https://www.keysight.com/us/en/assets/7018-06736/technical-overviews/5966-3063.pdf.

[7] Kitamura Y., Matoba M., Misawa T., Unesaki H., Shiroya S. (1999). Reactor noise experiments by using acquisition system for time series data of pulse train,. J. Nucl. Sci. Technol..

